# Pro-apoptotic and cell cycle-modulating effects of lobaric and rhizocarpic acids in human leukemic cell lines

**DOI:** 10.1007/s11033-026-12179-x

**Published:** 2026-06-23

**Authors:** Fabrizia Brisdelli, Alessandra Piccirilli, Benedetta Cinque, Giuseppe Celenza, Mariagrazia Perilli

**Affiliations:** 1 Department of Biotechnological and Applied Clinical Sciences, University of L’Aquila, L’Aquila, Italy; 2 Department of Life, Health and Environmental Sciences, University of L’Aquila, L’Aquila, Italy

**Keywords:** Apoptosis, Lobaric acid, Rhizocarpic acid, Lichen secondary metabolites, K562 cells, MOLM-6 cells

## Abstract

**Background:**

Lobaric and rhizocarpic acids are secondary metabolites isolated from Antarctic lichens. While several biological activities have been described for lobaric acid, the cellular effects of rhizocarpic acid remain poorly characterized. This study investigated the impact of these compounds on proliferation, apoptosis, and cell cycle regulation in human leukemic cell models.

**Methods and Results:**

Human leukemic K562 and MOLM-6 cells were exposed to lobaric and rhizocarpic acids under defined experimental conditions; cell viability, apoptosis, cell cycle distribution, and protein expression were evaluated using cell proliferation assays, flow cytometry, and western blot analysis. Both metabolites reduced cell growth and promoted caspase-dependent apoptosis in K562 and MOLM-6 cells. At the molecular level, in K562 cells, lobaric acid treatment was associated with increased Bax protein expression, whereas rhizocarpic acid induced upregulation of Bcl-2. In MOLM-6 cells, both compounds decreased STAT5 protein expression. In addition, both metabolites altered cell cycle distribution. Lobaric acid caused a significant increase of K562 cells in the G_2_/M phase and a transient rise of MOLM-6 cells in G_1_ phase, whereas rhizocarpic acid increased the proportion of cells in the G_1_ phase, with a concomitant reduction in S and G_2_/M populations in both leukemic cell lines. Analysis of cell cycle regulatory protein expression showed modulation of cyclin B1, cyclin D3, CDK4, and CDK6 by rhizocarpic acid, and upregulation of cyclin D3 by lobaric acid in both cell lines.

**Conclusions:**

Overall, these findings provide new insights into the molecular mechanisms underlying the bioactivity of lichen-derived metabolites in leukemic cells and identify rhizocarpic acid as a previously uncharacterized modulator of apoptosis- and cell cycle-related pathway progression. Further studies, including comprehensive dose-response analyses and *in vivo* evaluations, will be necessary to fully define their therapeutic potential.

**Supplementary Information:**

The online version contains supplementary material available at 10.1007/s11033-026-12179-x.

## Introduction

Natural products represent a useful source of molecules with several biological activities that can find applications in medicine. Lichens have been traditionally used for treating several disorders without being associated with any adverse effects [1].

Lichens are complex symbiotic associations between fungi and algae. This symbiosis results in the biosynthesis of secondary metabolites, that are unique in nature.

The most common classes of these lichen compounds are depsides, depsidones, usnic acid, dibenzofurans, xanthones, anthraquinones, pulvinic acid derivatives, and aliphatic acids [2]. The biosynthesis of secondary metabolites is not essential for lichen growth, but it depends on several environmental factors, such as UV exposure, temperature, altitude, and seasonality [3].

Due to the presence of unique substances in lichens, the potential medical applications of lichens have attracted many researchers over the years. Lichen secondary metabolites have shown a wide spectrum of biological and pharmacological properties, such as antimicrobial, analgesic, anti-inflammatory, anti-proliferative, and cytotoxic activities [4, 5].

Lobaric acid, a depsidone, and rhizocarpic acid, a pulvinic acid derivative, are two metabolites isolated from Antarctic lichens; while several interesting biological activities have been associated with lobaric acid, rhizocarpic acid has been poorly investigated.

Lobaric acid has demonstrated anti-cancer activity toward several cancer types, including glioma (U87MG cells) [6], cervical adenocarcinoma (HeLa cells), colon carcinoma (HCT116 cells) [7, 8], breast cancer (T-47D and ZR-75-1 cells), and leukemia (K562 cells) [9, 10]. Importantly, it induced only mild cytotoxicity in the normal cell line NIH-3T3 (mouse embryonic fibroblasts), with IC_50_ values higher than those observed in cancer cell lines [7].

Lobaric acid inhibits cell proliferation through cell cycle arrest and induction of apoptosis via the mitochondrial apoptotic pathway in HeLa and HCT116 cells [8]. Since altered cell cycle regulation plays a central role in tumor progression, identifying molecules that target cell cycle checkpoints may represent a promising therapeutic strategy because, in addition to suppressing cell proliferation, they could sensitize cancer cells to combination treatments [11].

However, the mechanisms underlying the cytotoxic activity of lobaric acid have not yet been well characterized in other types of cancer cells, especially leukemic cells.

Rhizocarpic acid is able to form complexes with Cu^2+^, Fe^2+^, Fe^3+^, Mg^2+^, Mn^2+^ and Zn^2+^, in both basic and acidic environments, suggesting a role in the metal ion homeostasis of lichens [12]. Its biological activity has been poorly studied. A mild antibacterial activity against *Bacillus subtilis* and *Staphylococcus aureus* and a modest cytotoxic activity against the mouse myeloma cell line NS-1 have been described [13, 14].

The purpose of this study was to investigate the antiproliferative and cytotoxic effects of lobaric acid and rhizocarpic acid on two Philadelphia chromosome-positive chronic myeloid leukemia (CML) cell lines, K562 and MOLM-6, representing different blast phases of CML. By assessing STAT5 and Bcl-2 family protein expression, apoptosis induction, and alterations in cell cycle progression, this work aims to evaluate the mechanism of action of these compounds. They may represent a promising strategy to overcome resistance to conventional BCR-ABL-targeted therapies, particularly when used in combination with tyrosine kinase inhibitors.

CML is a neoplasm characterized by a (9;22)(q34;q11) translocation that gives rise to the BCR-ABL chimeric protein [15]. BCR-ABL is a constitutively active tyrosine kinase that, in turn, drives multiple survival and proliferation signaling pathways [16].

Although inhibitors of BCR-ABL kinase are currently used to treat CML, resistance to these drugs renders the therapies less effective. Several natural products have exhibited anti-CML activity [17] and some, such as curcumin, can suppress the proliferation of K562 cells and enhance the efficacy standard therapies such as imatinib mesylate by downregulating the expression of several key molecular markers, including p210 BCR-ABL, Hsp90, survivin, and NF-κB subunits p65 and p50 [18].

## Materials and methods

### Materials

RPMI 1640 medium, penicillin, streptomycin, glutamate fetal bovine serum, nonfat dry milk were from Euroclone. Acridine orange, trypan blue, Nonidet-P40, EGTA, EDTA, dithiothreitol (DTT), Triton X-100, ethanol, isopropanol, dimethyl sulfoxide (DMSO), propidium iodide, RNase, MTT, a cocktail of protease inhibitors and ethidium bromide were purchased from Sigma Chemical Co (St. Louis, MO, USA). Anti-actin (I-19), anti-tubulin (D-10), anti-Bcl-2 (100), anti-Bax (N-20), anti-STAT5 (A-9), and anti-cyclin B1(GNS1) antibodies, diluted 1:200, were purchased from Santa Cruz Biotechnology (Santa Cruz, CA, USA). Anti-cyclin D3 (DCS22), anti-CDK4 (D9G3E) and anti-CDK6 (DCS83), diluted 1:1000, were from Cell Signaling Technology (Massachusetts, MA, USA). Peroxidase-conjugated secondary IgG antibodies were from Thermo Scientific Inc. (Hudson, NH, USA). Reagents for enhanced chemiluminescence (ECL) detection were obtained from Advansta (San Jose, CA, USA). Fluorogenic caspase substrates Ac-DEVD-AMC (acetyl-Asp-Glu-Val-Asp-aminomethylcoumarin), Ac-IETD-AFC (acetyl-Ile-Glu-Thr-Asp-aminotrifluoromethylcoumarin) and Ac-LEHD-AMC (acetyl-Leu-Glu-His-Asp-aminomethylcoumarin) were from Alexis Biochemicals (San Diego, CA, USA). All other chemicals were of reagent grade. Stock solutions of lobaric acid and rhizocarpic acid were prepared in DMSO.

### Lichen compounds

Lobaric acid and rhizocarpic acid, donated by Prof. Marisa Piovano (Universidad Técnica Federico Santa María, Valparaíso, Chile), were isolated from a chloroform extract of *Stereocaulon alpinum* collected during January and February in Ardley Cove, King George Island, South Shetland Islands, Antarctica, and from an acetone extract of *Rhizocarpon geographicum* collected during January and February in Coppermine Cove, Robert Island, South Shetland Islands, Antarctica, respectively, by elution on a silica gel column. The degree of purity was > 98% as determined by thin layer chromatography and ^1^H-NMR analyses [19, 20]. The structures of the compounds studied are presented in Figure S1.

### Cell culture

The human chronic myelogenous leukemia K562 and MOLM-6 cell lines were obtained from the American Type Culture Collection (ATCC) and the DSMZ Culture Collection, respectively. They were grown in RPMI 1640 medium supplemented with 10% heat-inactivated fetal bovine serum, 100 U/mL penicillin, 100 µg/mL streptomycin, and 2 mM L-glutamine. Cells were maintained at 37 °C in a humidified 5% CO_2_ atmosphere. For experiments, K562 cells were seeded at a density of 2 × 10^5^/mL, whereas MOLM-6 cells were seeded at a density of 5 × 10^5^/mL. After 24, 48 and 72 h, cells were counted using an inverted microscope (Telaval 31, Zeiss, Germany), and viability was determined by the trypan blue exclusion assay. The percentage of viable cells was calculated according to the following formula:

% of viable cells = (number of total cells - number of blue cells) / number of total cells x 100.

### MTT assay

The MTT colorimetric method was performed as described previously [21]. Cells were exposed to increasing concentrations of compounds for 48 h. The same amount of DMSO was added to untreated controls. Then, MTT solution was added to each well, at a final concentration of 0.5 mg/mL, for 3 h at 37 °C. After solubilization of the formazan crystals by the addition of acidified isopropanol (0.04 N HCl in isopropanol), the absorbance at 570 nm was measured on a microplate reader (Infinite M Plex, Tecan). The viability of untreated cells was considered 100% and cell survival was determined by comparing the absorbance of treated and untreated cells.

### Apoptosis evaluation

Nuclear morphology was assessed by acridine orange/ethidium bromide double staining assay. Cells were washed with PBS, stained with a fluorescent solution containing 100 µg/mL ethidium bromide and 100 µg/mL acridine orange in PBS, and immediately observed with a fluorescence microscope (Dialux 20, Leitz, Germany). About 400 cells were analyzed for each determination. Green clumped nuclei indicated chromatin condensation with intact membrane structures (early apoptosis); orange cells with clumped nuclei indicated later apoptosis. Nuclei of necrotic cells appeared uniformly stained by EB. The percentage of apoptotic cells was calculated according to the following formula:

% of apoptotic cells = (number of early apoptotic cells + number of late apoptotic cells) / number of total cells x 100.

### Caspase activity

Cells were washed with PBS and then lysed in a buffer containing 50 mM Tris-HCl, pH 7.4, 10 mM EGTA, 1 mM EDTA, 10 mM DTT, 1% (v/v) Triton X-100, for 30 min at 4 °C. The lysed cells were centrifuged at 15,000 × g for 15 min at 4 °C; the supernatants were collected and used for detection of caspase activity. Protein concentrations were determined by Bradford method [22] using Biorad protein assay dye reagent concentrate (Bio-Rad Laboratories, Milano Italy). Cell lysates (60 µg of proteins) were mixed in the reaction buffer (50 mM Tris–HCl, pH 7.4, 10 mM EGTA, 1 mM EDTA, 10 mM DTT) with 20 µM fluorogenic caspase peptide substrates, Ac-DEVD-AMC (for caspase-3) for 30 min, Ac-IETD-AFC (for caspase-8) and Ac-LEHD-AMC (for caspase-9) for 1 h, at 37 °C. Fluorescence was measured on a Perkin-Elmer LS-50B spectrofluorometer, with excitation at 380 nm and emission at 460 nm for caspase-3 and 9 activities and with excitation at 400 nm and emission at 505 nm for caspase-8 activity.

### Cell cycle analysis by flow cytometry

Cells were collected, washed twice with ice-cold PBS and fixed in 70% ethanol at 4 °C for 30 min. The fixed cells were then washed twice with ice-cold PBS and stained with a solution containing 50 µg/mL propidium iodide, 0.1% Nonidet-P40 and 25 µg/mL RNase. Cell cycle phase distribution was analyzed using flow cytometry (FACScan flow cytometry, Becton-Dickinson Immunocytometry System, San Jose, CA, USA). Data from 10,000 events per sample were collected and analyzed using the cell cycle analysis software (Modfit LT for Mac V 3.0) to calculate the percentages of cells in the G_1_, G_2_/M and S phases.

### Western blotting analysis

Cells were collected after treatments, washed with PBS and lysed in RIPA buffer containing a suitable cocktail of protease inhibitors, for 30 min at 4 °C. Proteins were separated by SDS-PAGE and transferred to polyvinylidene difluoride (PVDF) membranes. Membranes were blocked with 5% (w/v) nonfat dry milk and immunoblotted with suitably diluted primary antibodies overnight at 4 °C, followed by secondary antibodies conjugated with horseradish peroxidase, used at 1:20000, for 1 h at room temperature. Bands were visualized using a chemiluminescent detection system (ChemiDoc XR plus, Bio-Rad Laboratories, Milano, Italy), quantified by ImageJ software (version 1.44; NIH, Bethesda, MD, USA) and normalized using an internal reference.

### Statistical analysis

Data are reported as mean ± SD (standard deviation). Statistical differences were calculated using the Student’s t test. Results were considered statistically significant at p-value ≤ 0.05.

## Results

### Cell growth and viability

**Fig. 1:**
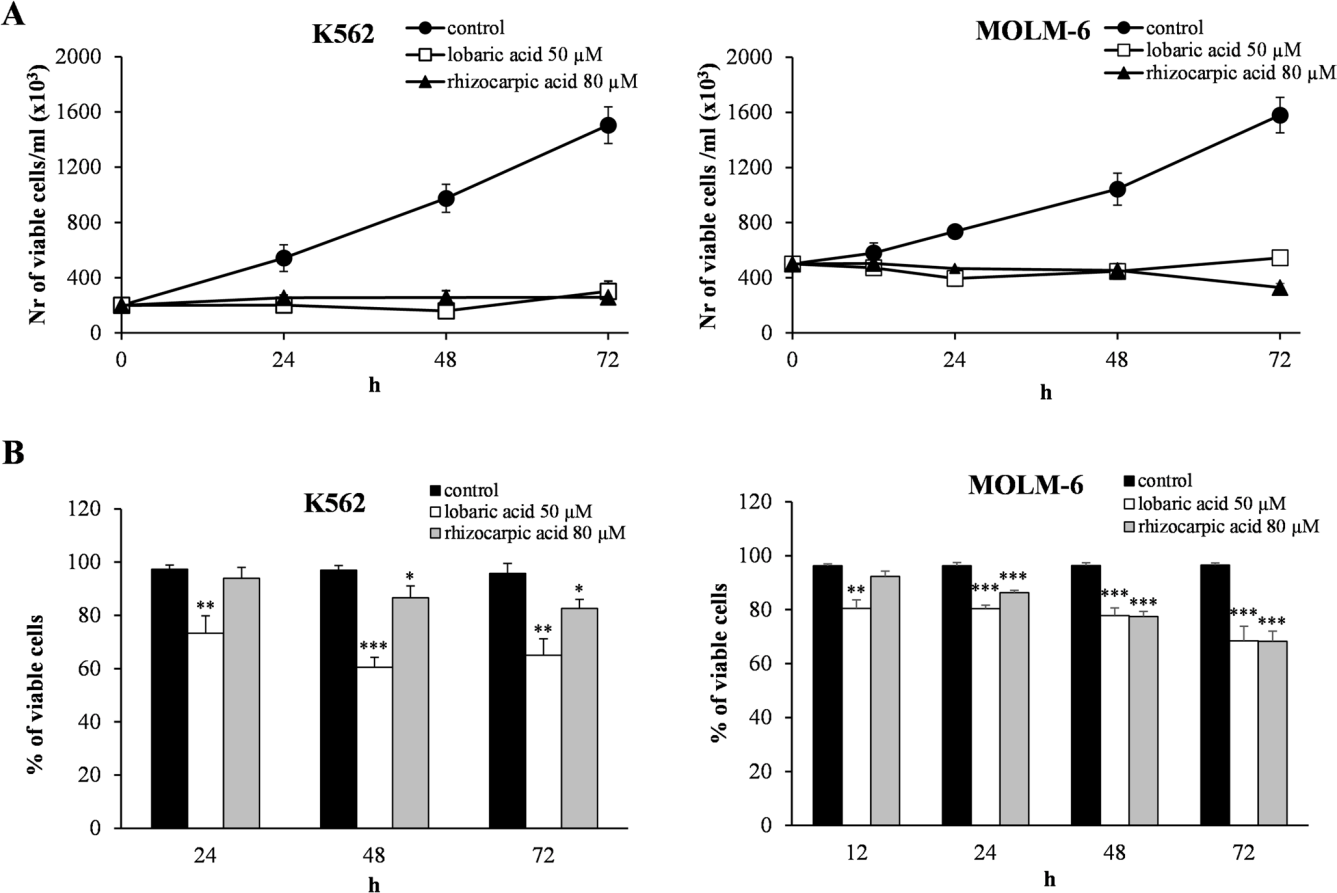
Effect of 50 µM lobaric acid and 80 µM rhizocarpic acid on K562 and MOLM-6 cell growth (**A**) and cell viability (**B**). Control: untreated K562 or MOLM-6 cells. Viability values are reported as absolute percentages. Results represent the mean ± SD of three independent experiments. *p < 0.05, **p < 0.01 and ***p < 0.001 compared to the untreated control

In preliminary MTT experiments performed in K562 cells, increasing concentrations (20–80 µM) of lichen metabolites were tested to identify exposure conditions capable of inducing measurable cytotoxic effects while preserving sufficient cell viability for subsequent mechanistic analyses. Based on these preliminary experiments, for further studies, K562 cells were exposed to 50 µM lobaric acid and 80 µM rhizocarpic acid, concentrations that reduced viability by approximately 50% and 30%, respectively (Fig. S2). These concentrations were applied to another leukemic cell line, MOLM-6, to evaluate cellular responses under identical exposure conditions. Cell growth and viability, evaluated by the trypan blue exclusion test, were determined after 24, 48, and 72 h of treatment. Under the tested conditions, both metabolites reduced cell proliferation after 24 and 48 h of exposure in both K562 and MOLM-6 cells. Notably, after rhizocarpic acid treatment, K562 cell numbers remained comparable to the initial seeding density, a pattern consistent with a predominantly cytostatic effect rather than a cytotoxic one. In lobaric acid-treated cultures, a partial recovery in viable cell numbers was observed at 72 h (Fig. 1A). In K562 cells, rhizocarpic acid treatment maintained more than 80% viability at all exposure times, with 17.4% trypan blue-positive cells after 72 h, whereas lobaric acid exposure resulted in 60.5% viable cells (39.5% trypan blue-positive cells) as early as 48 h of treatment (Fig. 1B). In MOLM-6 cells, both compounds induced approximately 32% trypan blue-positive cells after 72 h of exposure.

Importantly, since independent dose-response curves and IC₅₀ determinations were not performed for each cell line, these data reflect cellular responses under fixed exposure conditions, specifically chosen to allow a comparison between the two models of the same disease under identical experimental settings, rather than to establish their relative drug sensitivity.

### Effect of lobaric and rhizocarpic acids on cell cycle distribution

In order to investigate the mechanisms involved in growth inhibition induced by lichen secondary metabolites, we evaluated whether the compounds could induce cell cycle arrest in K562 and MOLM-6 cells using flow cytometric analyses.

**Fig. 2:**
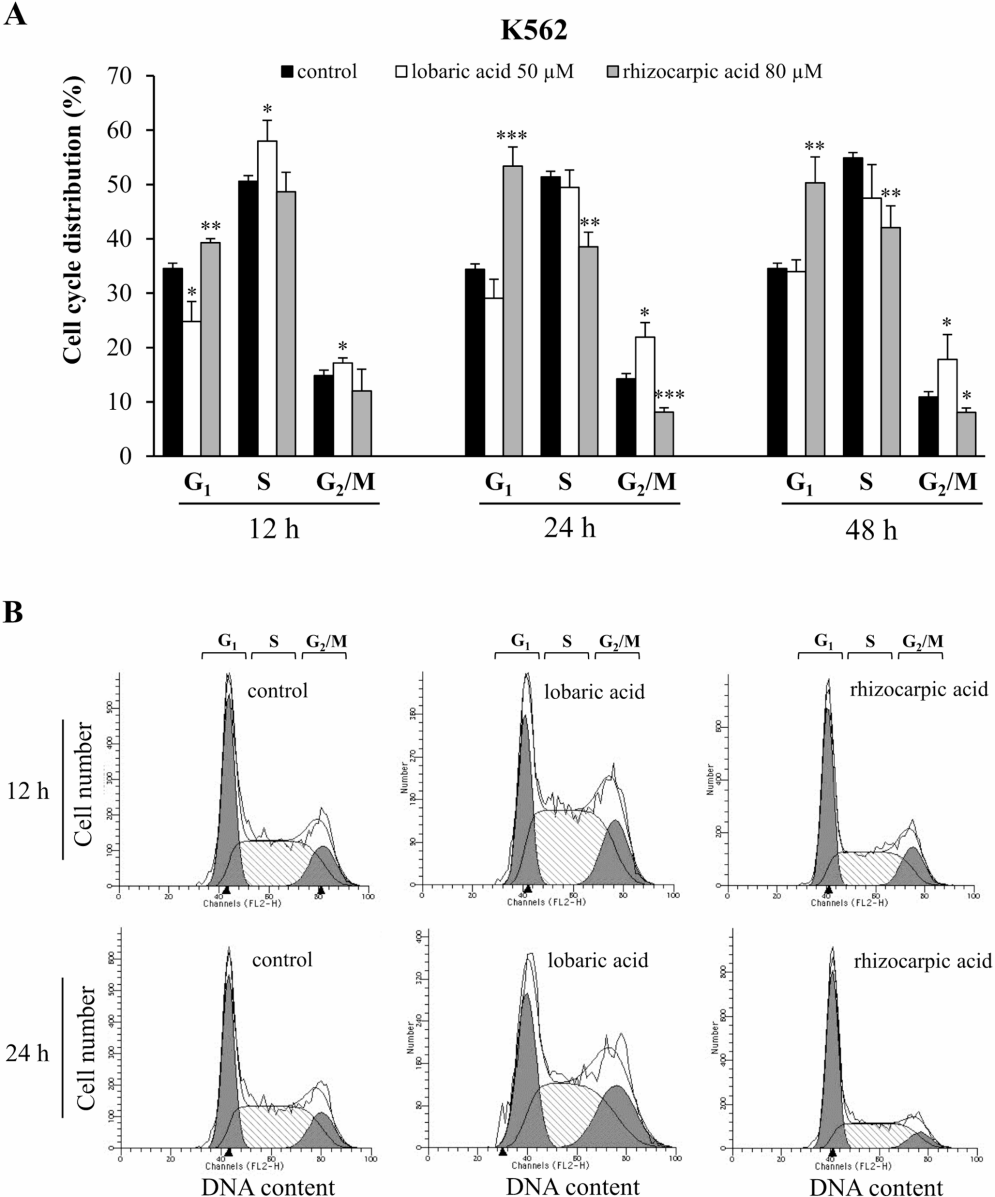
Effect of 50 µM lobaric acid and 80 µM rhizocarpic acid on cell-cycle progression of K562 cells. (**A**) Cell cycle analysis by propidium iodide staining and flow cytometry after indicated treatments for 12, 24 and 48 h. Control: untreated K562 cells. Results represent the mean ± SD of three independent experiments. *p < 0.05, **p < 0.01 and ***p < 0.001 compared to the untreated K562 cells. (**B**) A pattern representative of three similar profiles of cell cycle distribution in K562 cell population. The profiles related to treatment times with the most representative modulation of the cell cycle were shown

**Fig. 3:**
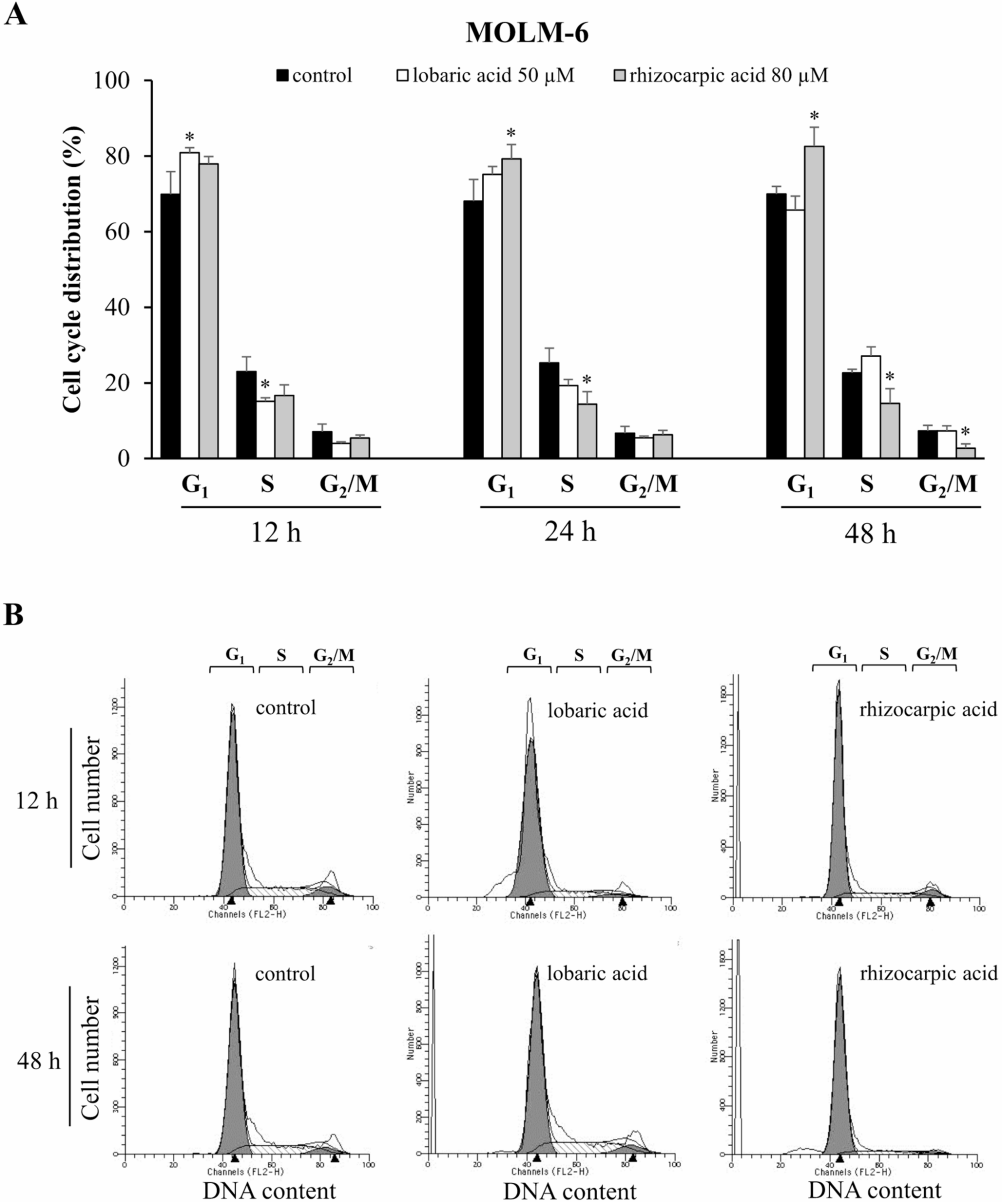
Effect of 50 µM lobaric acid and 80 µM rhizocarpic acid on cell-cycle progression of MOLM-6 cells. (**A**) Cell cycle analysis by propidium iodide staining and flow cytometry after indicated treatments for 12, 24 and 48 h. Control: untreated MOLM-6 cells. Results represent the mean ± SD of three independent experiments. *p < 0.05, **p < 0.01 and ***p < 0.001 compared to the untreated MOLM-6 cells. (**B**) A pattern representative of three similar profiles of cell cycle distribution in MOLM-6 cell population. The profiles related to treatment times with the most representative modulation of the cell cycle were shown

Rhizocarpic acid-treated K562 and MOLM-6 cells showed a cell cycle profile significantly different from that of the untreated control cells (Figs. 2 and 3). An increase in the percentage of cells in the G_1_ phase in association with a decrease in the percentage of cells in the S and G_2_/M phase was observed after 24 and 48 h of treatment. Lobaric acid induced a progressive increase in the number of cells in the G_2_/M phase from 12 h of treatment to 48 h in K562 cells (Fig. 2A and B), while in MOLM-6 cells, after 12 h of treatment, an increase in the number of cells in the G_1_ phase, in association with a decrease of cells in the S phase, was observed (Fig. 3A and B). After 24 and 48 h, the cell cycle profiles of lobaric acid-treated and untreated MOLM-6 cells became similar.

To further confirm the observed cell cycle alterations, the levels of several essential cell cycle regulatory proteins were analyzed after 24 h of treatments using the western blotting method.

**Fig. 4:**
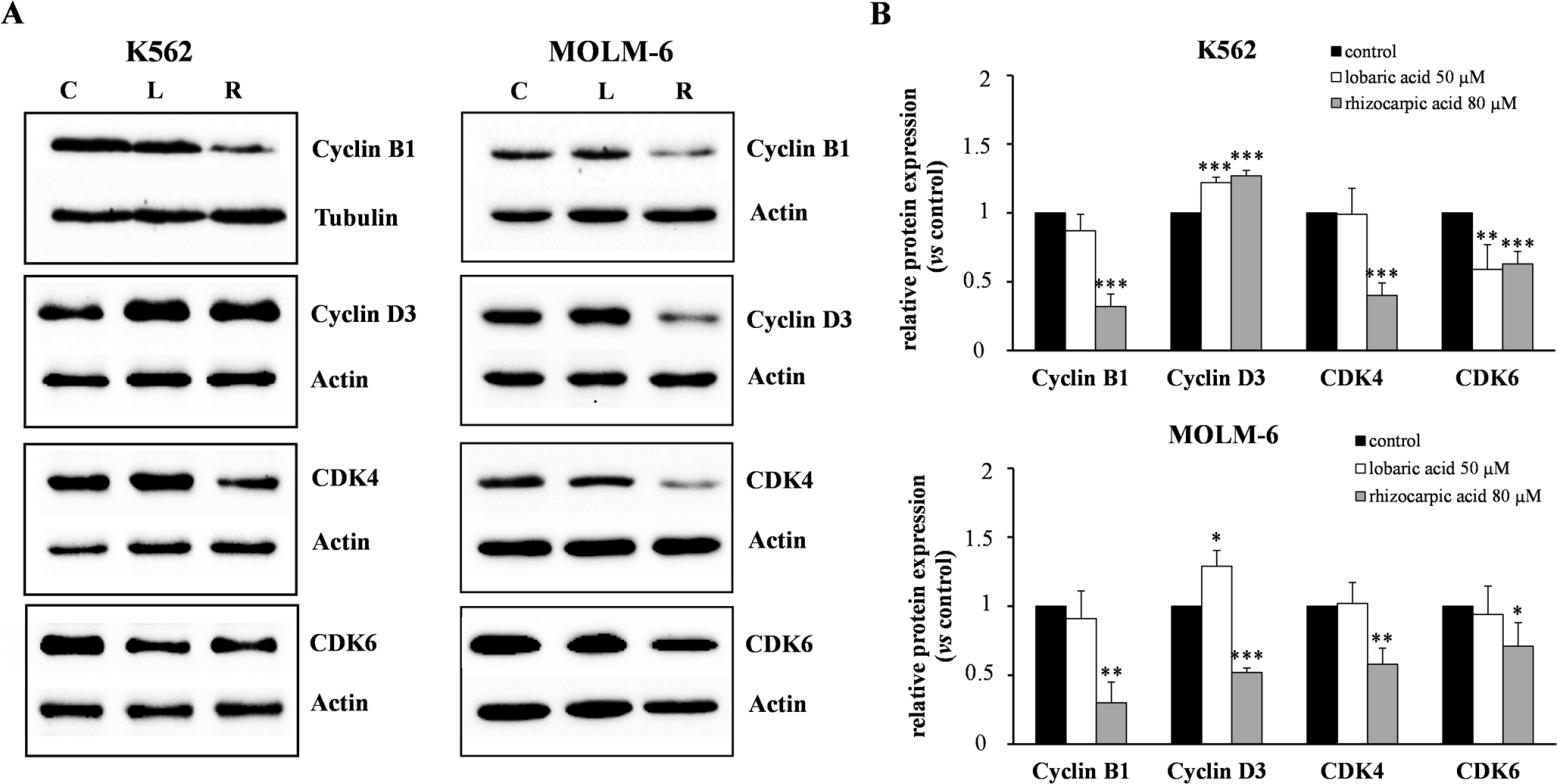
The protein content of Cyclin B1, Cyclin D3, CDK4 and CDK6 was analyzed by western blotting in K562 and MOLM-6 cells treated for 24 h with 50 µM lobaric acid and 80 µM rhizocarpic acid. (**A**) Representative immunoblots of three independent experiments with similar results; C: control, untreated K562 or MOLM-6 cells; L: lobaric acid-treated cells; R: rhizocarpic acid-treated cells. (**B**) In the densitometric analysis, after normalization with actin or tubulin protein levels, relative protein expression values have been determined as ratios between the intensities of protein bands in treated and untreated cells, assigning the value 1 to the untreated control. Results represent the mean ± SD of three independent experiments. *p < 0.05, **p < 0.01 and ***p < 0.001 compared to the untreated cells

Rhizocarpic acid treatment induced a significant decrease, compared to untreated cells, in the expression levels of cyclin B1, CDK4 and CDK6 in both K562 and MOLM-6 cells, while, regarding the expression of cyclin D3, it showed a different behavior in the two cell lines. In K562 cells, rhizocarpic acid induced an upregulation of cyclin D3, whereas in MOLM-6 cells a decrease of cyclin D3 was also observed (Fig. 4). Lobaric acid induced an increase in cyclin D3 levels in both K562 and MOLM-6 cells and was also able to modulate CDK6 expression, downregulating it only in K562 cells. No significant differences, compared to untreated control cells, were detected for cyclin B1 and CDK4 in K562 and MOLM-6 cells and for CDK6 in MOLM-6 cells treated with lobaric acid (Fig. 4).

### Effect of lobaric and rhizocarpic acids on apoptosis in K562 and MOLM-6 leukemic cells

Nuclear morphology was examined by fluorescence microscopy to determine whether the observed decrease in cell viability, following lobaric and rhizocarpic acid treatments, was due to apoptosis.

**Fig. 5:**
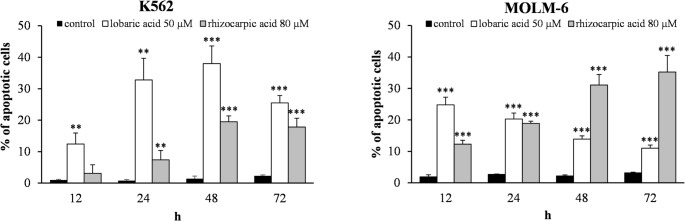
Effect of 50 µM lobaric acid and 80 µM rhizocarpic acid on K562 and MOLM-6 cell apoptosis. Analysis of characteristic nuclear morphological features of apoptosis. The percentage of condensed and fragmented nuclei was estimated, by fluorescence microscope, on acridine orange and ethidium bromide double-stained cells, examined at the indicated times. Control: untreated K562 or MOLM-6 cells. Results represent the mean ± SD of three independent experiments. *p < 0.05, **p < 0.01 and ***p < 0.001 compared to the untreated control

As shown in Fig. 5, lobaric acid treatment induced a time-dependent increase in apoptotic features in K562 cells, reaching 38% apoptotic cells after 48 h of incubation, followed by a decline at 72 h. This decrease in apoptosis correlates with the observed partial recovery of proliferation at later time points, likely due to surviving cells resuming proliferation (Fig. 1A).

Rhizocarpic acid caused less chromatin condensation in K562 cells, reaching only 19.5% apoptotic cells after 48 h of exposure, a value that remained stable up to 72 h (Fig. 5).

In MOLM-6 cells, rhizocarpic acid treatment was associated with a time-dependent increase in apoptotic cells, reaching approximately 35% after 72 h of treatment (Fig. 5).

Lobaric acid exerted an early increase in apoptotic cells (24.8% at 12 h), followed by a decline at prolonged incubation times (Fig. 5).

### Evaluation of caspase-mediated apoptosis

The distinct kinetics and extent of apoptotic morphological changes observed under the selected treatment conditions, were further evaluated by spectrofluorimetric analysis of caspase-3, -8, and - 9 activities.

**Fig. 6:**
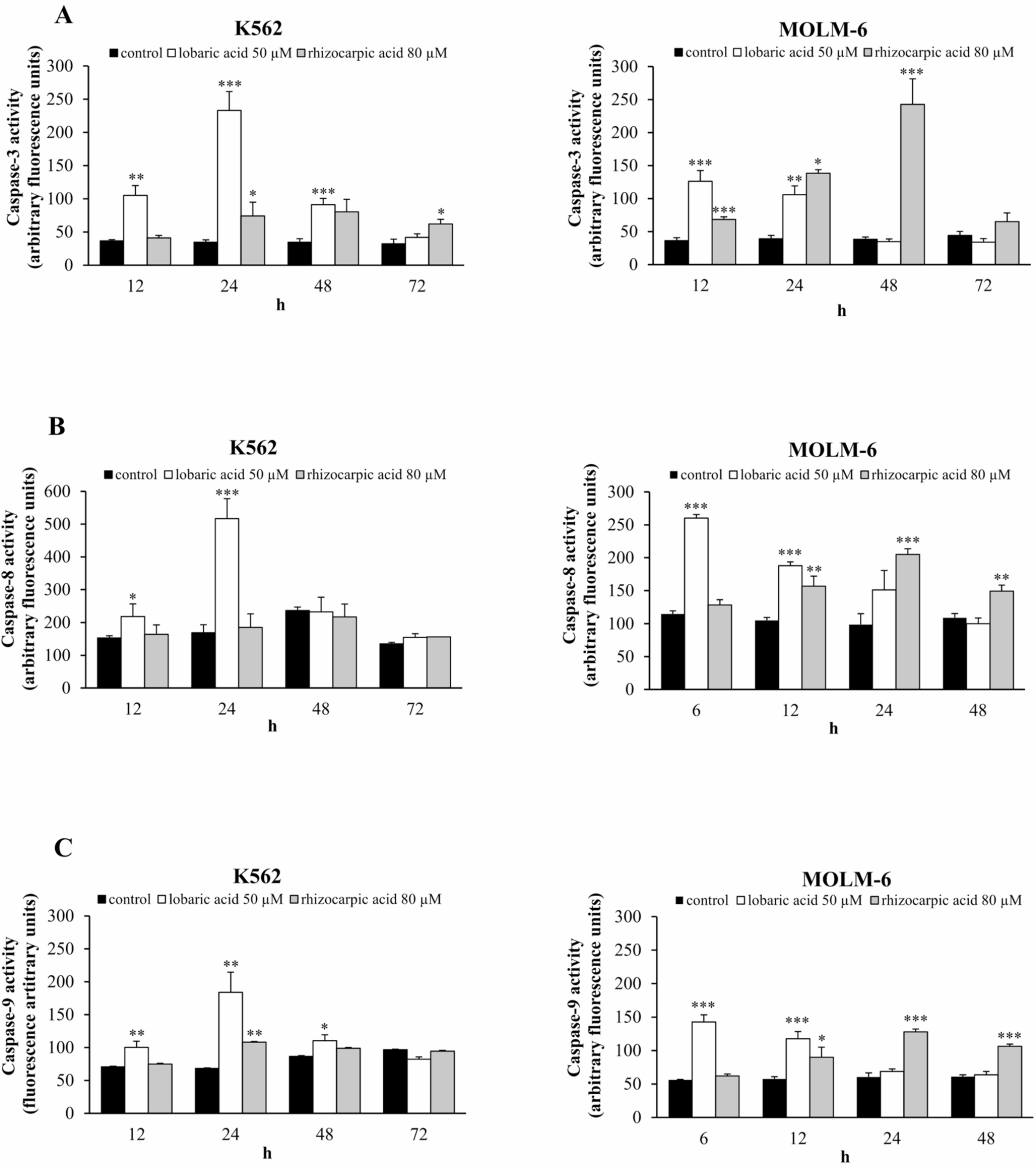
Time course of caspase-3 (A), -8 (B) and  -9 (C) activities in K562 and MOLM-6 cells treated with 50 µM lobaric acid and 80 µM rhizocarpic acid. Control: untreated K562 or MOLM-6 cells. Results represent the mean ± SD of three independent experiments. *p < 0.05, **p < 0.01 and ***p < 0.001 compared to the untreated control

In lobaric acid-treated K562 cells, caspase-3 activity significantly increased from 12 h to 24 h, reaching a 6.7-fold increase relative to untreated cells, at 24 h of treatment, and subsequently returning to baseline levels after 72 h (Fig. 6A). In lobaric acid-treated MOLM-6 cells, a more modest caspase-3 activation (3.5-fold as compared to untreated cells) was observed at 12 h, followed by a decline to basal levels already after 48 h (Fig. 6A).

Rhizocarpic acid treatment resulted in a mild (about twofold) and non-progressive increase in caspase-3 activity in K562 cells, from 24 h to 72 h, whereas in MOLM-6 cells it induced a progressive increase in caspase-3 activity, reaching a maximum of 6.3-fold over control levels after 48 h (Fig. 6A).

To further examine the mechanisms triggered by lobaric and rhizocarpic acids, the activation of initiator caspase-8 and caspase-9 was investigated. Since an earlier apoptotic response to lobaric acid observed in MOLM-6 cells, the caspase-8 and - 9 activities, preceding the activation of effector caspase-3, were assessed as early as 6 h. Lobaric acid induced a significant increase in both caspase-8 and caspase-9 activities in both cell lines (Fig. 6B and C). In contrast, rhizocarpic acid induced activation of both initiator caspase-8 and  -9 only in MOLM-6 cells, while in K562 cells, only caspase-9 activation was detected (Fig. 6B and C).

### Effect of lobaric and rhizocarpic acids on Bax, Bcl-2 and STAT5 expression

**Fig. 7:**
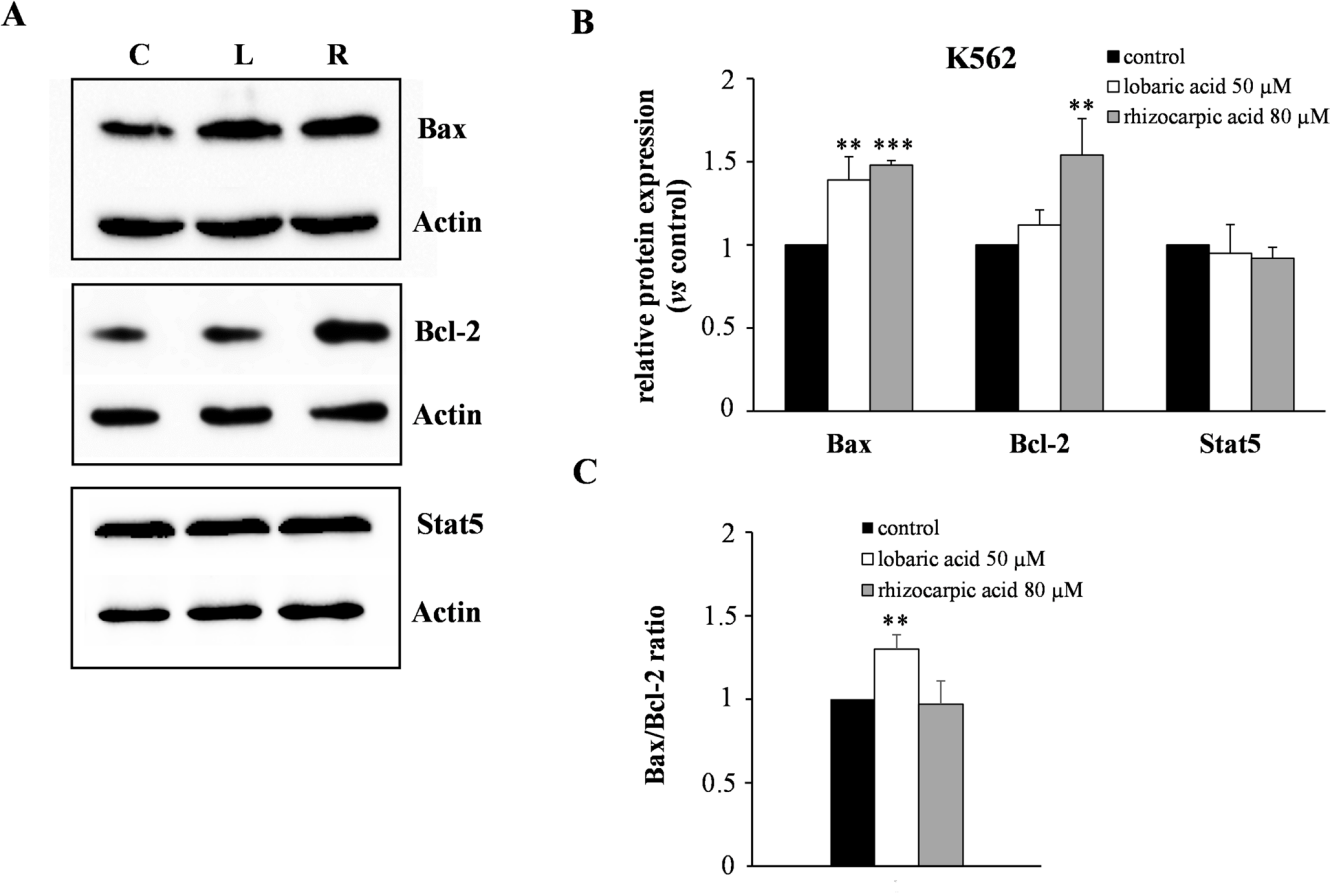
The protein content of Bax, Bcl-2 and Stat5 was analyzed by western blotting in K562 cells treated for 24 h with 50 µM lobaric acid and 80 µM rhizocarpic acid. (**A**) Representative immunoblots of three independent experiments with similar results; C: control, untreated K562 cells; L: lobaric acid-treated cells; R: rhizocarpic acid-treated cells. (**B**) In the densitometric analysis, after normalization with actin protein levels, relative protein expression values have been determined as ratios between the intensities of protein bands in treated and untreated cells, assigning the value 1 to the untreated control. (**C**) Effect of lobaric and rhizocarpic acids on the Bax/Bcl-2 ratio. Results represent the mean ± SD of three independent experiments. *p < 0.05, **p < 0.01 and ***p < 0.001 compared to the untreated K562 cells.

**Fig. 8:**
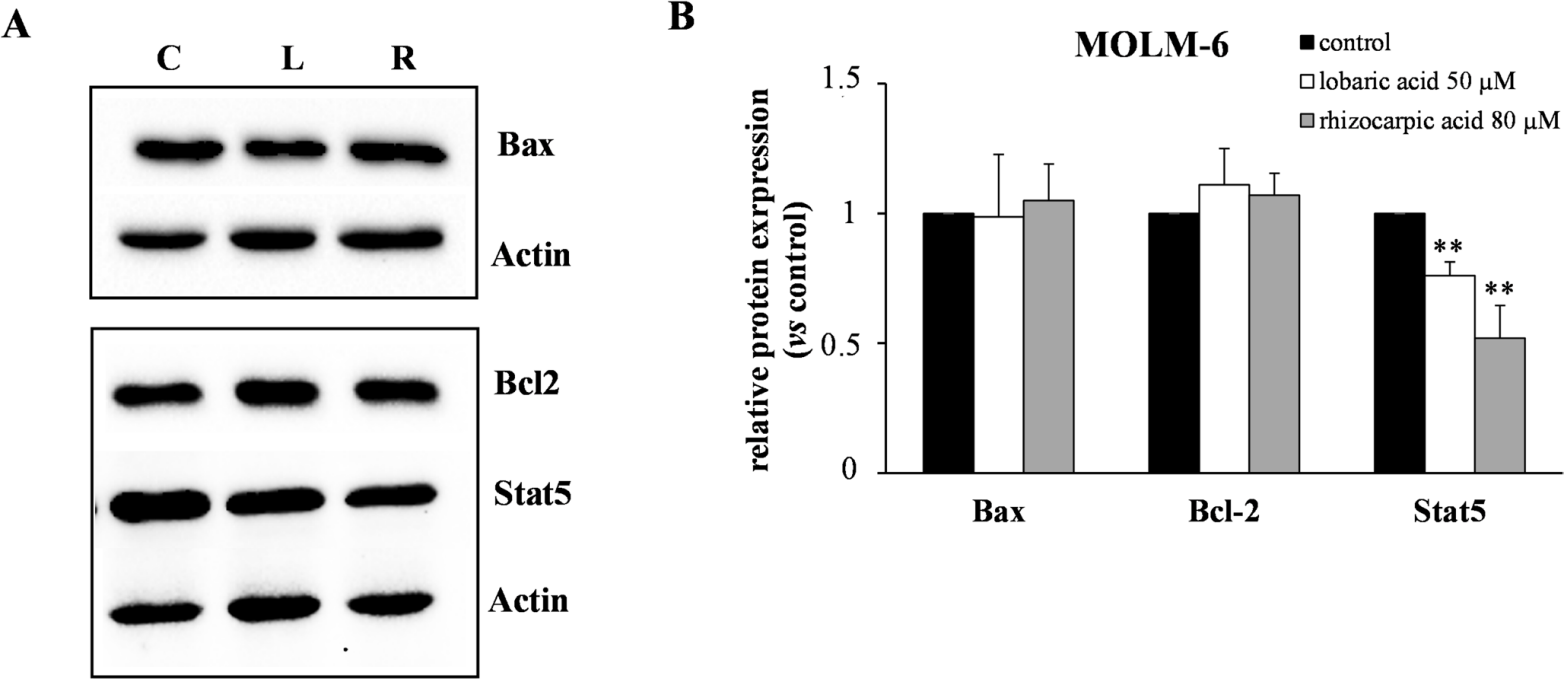
The protein content of Bax, Bcl-2 and Stat5 was analyzed by western blotting in MOLM-6 cells treated for 24 h with 50 µM lobaric acid and 80 µM rhizocarpic acid. (**A**) Representative immunoblots of three independent experiments with similar results; C: control, untreated MOLM-6 cells; L: lobaric acid-treated cells; R: rhizocarpic acid-treated cells. (**B**) In the densitometric analysis, after normalization with actin protein levels, relative protein expression values have been determined as ratios between the intensities of protein bands in treated and untreated cells, assigning the value 1 to the untreated control. Results represent the mean ± SD of three independent experiments. *p < 0.05, **p < 0.01 and ***p < 0.001 compared to the untreated MOLM-6 cells

To explore the mechanism of apoptosis-mediated cell death, the expression of apoptosis-related proteins, Bcl-2 and Bax, was analyzed by western blotting after 24 h of treatments. Both metabolites significantly increased the expression of Bax protein in K562 cells, whereas they showed different effects on the expression of Bcl-2: it remained unchanged after exposure to lobaric acid, while it was upregulated by rhizocarpic acid (Fig. 7A and B). Then, in K562 cells, the Bax/Bcl-2 ratio was increased by lobaric acid, whereas this ratio did not undergo significant changes following rhizocarpic acid treatment, since both proteins were overexpressed (Fig. 7C). In MOLM-6 cells, the expression of Bax and Bcl-2 proteins was not modulated (Fig. 8A and B).

Since STAT5 is a signal transduction protein constitutively active in many forms of hematologic cancers, including chronic myelogenous leukemia, its expression was also analyzed by western blotting after 24 h of treatments. Lobaric and rhizocarpic acids induced a significant downregulation of STAT5 protein in MOLM-6 (Fig. 8A and B). Nonetheless, rhizocarpic acid showed a more marked effect on STAT5 expression than lobaric acid. No change in the expression of STAT5 was observed in treated K562 cells as compared with untreated controls (Fig. 7).

## Discussion

Lichens represent an interesting source of promising molecules for drug discovery and development. Several lichen-derived secondary metabolites have shown antiproliferative and cytotoxic properties against different cancer models, suggesting that these natural products may represent promising scaffolds for the development of new anticancer agents [23]. In particular, metabolites, such as usnic acid and its derivatives, lobaric acid, hypostictic acid and salazinic acid have demonstrated cytotoxic activity towards leukemic cell lines [5, 9].

Their biological activity has been often associated with the modulation of oxidative stress, interference with cell cycle progression, and activation of apoptotic pathways.

In this context, the effects observed in the present study are consistent with the emerging role of lichen metabolites as bioactive compounds with potential anticancer properties, although the specific mechanisms may differ depending on the compound and cellular model.

This work investigates the biological effects of two lichen-derived metabolites, lobaric acid and rhizocarpic acid, in leukemic cell models and explored the molecular mechanisms underlying their antiproliferative activity. Overall, our findings suggest that both compounds inhibit leukemic cell growth through a mechanism involving modulation of cell cycle regulatory proteins, induction of cell cycle arrest, and activation of apoptotic pathways.

Specifically, alterated expression of key regulators of the G_1_/S and G_2_/M transitions, together with caspase activation and modulation of apoptosis-related proteins, suggest that interference with cell cycle progression represents a central event leading to programmed cell death.

To our knowledge, this is the first report describing the apoptotic activity of rhizocarpic acid in leukemic cells and further confirms the pro-apoptotic properties of lobaric acid also in leukemic models, extending previous observations obtained in other tumor cell systems.

The *in vitro* antiproliferative and cytotoxic activity of lobaric acid has been previously evaluated in many different cancer cell lines, including K562 cells, although only a limited number of molecular mechanisms have been identified, such as anti-apoptotic Bcl-2 protein modulation, oxidative DNA damage, inhibition of 5-lipoxygenase and 12-lipoxygenase, and tubulin polymerization inhibition [24]. In agreement with these observations, our results further demonstrate that lobaric acid can trigger caspase activation and modulate protein involved in cell cycle regulation and apoptotic pathways.

The two cell lines used, K562 and MOLM-6, represent distinct subtypes of CML blast phase, exhibiting erythroleukemic and myeloid characteristics, respectively, that may respond differently to antiproliferative compounds.

K562 cells have been extensively characterized at the molecular level, while MOLM-6 cells have been rarely employed in experimental research, and further studies are needed to better elucidate their biological and pharmacological features. Their intrinsic biological differences may influence the cellular response to lichen-derived metabolites and should therefore be considered when interpreting the differential effects observed in our experiments.

Both lobaric and rhizocarpic acids induced growth inhibition and apoptosis in K562 and MOLM-6 cells under the selected experimental conditions. However, the differences observed in the magnitude and kinetics of the apoptotic responses between the two cell lines should be interpreted cautiously. Since independent dose-response analyses and IC₅₀ determinations were not performed in MOLM-6 cells, the applied concentrations cannot be considered pharmacologically equivalent between the two cellular models. Therefore, the apparent differences in responsiveness likely reflect intrinsic biological variability rather than differences in relative drug sensitivity.

Furthermore, the apparent discrepancy between the decrease in apoptotic features over time and the continued reduction in MOLM-6 cell viability after lobaric acid treatment may be attributed to the dynamic progression of apoptosis and differences in assay sensitivity. Trypan blue exclusion detects loss of membrane integrity, which occurs at late stages of cell death, whereas morphological assessment captures both early and late apoptotic cells. In addition, apoptotic cells may fragment and form apoptotic bodies, potentially leading to an underestimation of apoptotic cells at later time points.

Since many antiproliferative agents preferentially affect actively cycling cells, differences in proliferative status may significantly influence cellular susceptibility to compounds that interfere with cell cycle progression. K562 cells exhibit a higher proliferation rate, with an approximate doubling time of 24 h, compared to MOLM-6 cells that display a doubling time ranging from 48 to 72 h.

Consistent with this hypothesis, lobaric acid treatment resulted in an accumulation of K562 cells in the G_2_/M phase of the cell cycle. Similar G_2_/M arrest has been reported in other cancer cell models, including HeLa and HCT116 cells [8], suggesting that lobaric acid may interfere with the G_2_/M checkpoint, potentially through the induction of genotoxic stress or mitotic dysfunction. Such interference may impair proper cell cycle progression and contribute to decreased cell viability. This increase in K562 cells in the G_2_/M phase was not associated with a significant modulation of most of the key proteins regulating cell cycle progression, analyzed in our study, including cyclin B1. Lobaric acid was able to downregulate only CDK6 expression in K562 cells.

CDK4/6 are structurally related cyclin D-dependent kinases, with biochemical and biological similarities, that promote the G_1_/S transition by the phosphorylation of retinoblastoma protein (RB), triggering transcription factor E2F release and transcription of E2F target genes [25].

Increasing evidence highlights the involvement of cyclins and CDKs in non-canonical roles such as regulating cell death, differentiation, DNA repair, metabolism and mitochondrial activity [26].

A wide variety of carcinomas and hematological malignancies are often characterized by hyperactivation of specific cyclin-CDK complexes that could be promising targets for an antitumoral strategy [27–29]. In several studies, it was observed that knockdown of CDK4 and CDK6 with shRNA or inhibitor treatments inhibited cell cycle progression and proliferation of CML cells, including K562 cells [30, 31], . While CDK6 activity promotes cell cycle progression, evidences indicate that CDK6 exerts other activities, some of which are kinase-independent, that may be important for the phenotype of leukemic cells [32].

Interestingly, rhizocarpic acid was able to downregulate the expression of both CDK4 and CDK6 proteins in both cell lines. These findings are particularly noteworthy in light of recent advances in the use of CDK4/6 inhibitors for the treatment of HR+/HER2- breast cancer [33].

MOLM-6 cells, which display a slower proliferation rate, showed a progressive apoptotic response to rhizocarpic acid treatment, which caused a G_1_/S transition slowdown in both leukemic cell lines. Whether proliferation rate directly contributes to this pattern remains to be clarified.

In addition to affecting CDKs, rhizocarpic acid downregulated cyclin B1 in both cell lines and cyclin D3 in MOLM-6 cells. Cyclin D3, together with its partner CDK4/6, operates in mid-to-late G1 phase to promote progression through the restriction point, and thus cell’s commitment to replicate its genome [34], whereas cyclin B1 is essential for mitotic entry, leading to the G_2_/M transition [35].

Cyclin B1 overexpression in several cancer cells [36] contributes to chromosomal instability by altering spindle checkpoint function [37], but it is typically absent in normal cells, making it a potential selective anticancer target.

Cyclin D3 upregulation was observed in both lobaric acid-treated K562 and MOLM-6 cells. This modulation of cyclin D3 may simply be linked to the resumption of the cell cycle observed after prolonged treatment, or it could alternatively play a role in influencing the apoptotic process, since cyclin D3 has shown both anti-apoptotic or pro-apoptotic roles, depending on the context. In leukemic T cells, ectopic cyclin D3 expression prevented apoptosis induced by phorbol myristate acetate (PMA) and T-cell receptor stimulation [34]. On the contrary, its overexpression sensitized HeLa and fibrosarcoma HT-1080 cells to TNF-induced apoptosis [38]. In HEK293 cells, cyclin D3 interacted with caspase-2, stimulating its activation and consequently inducing apoptosis [39].

Analysis of the expression of apoptosis-related proteins revealed that, unlike previous findings in Hela and HCT-116 cells [8], in our leukemia models, lobaric acid-induced apoptosis was not associated with decreased Bcl-2 protein expression.

In K562 cells, the more pronounced apoptosis observed with lobaric acid treatment was associated with upregulation of Bax protein and simultaneous activation of both caspase-8 and caspase-9, suggesting a cross-talk between the extrinsic and intrinsic pathways. In rhizocarpic acid-treated K562 cells, both the absence of caspase-8 activation and the upregulation of Bcl-2 protein, which also reduces the involvement of the mitochondrial pathway, could explain the reduced apoptotic effect.

In MOLM-6 cells, lobaric acid exhibited an earlier apoptotic activity, but was less effective as the effect was lost during prolonged incubation times and was not associated with modulation of the Bcl-2/Bax ratio.

Interestingly, both lobaric and rhizocarpic acids induced a selective downregulation of STAT5 protein in MOLM-6 cells; however, rhizocarpic acid showed a more marked effect that correlates with its stronger and more prolonged apoptotic activity.

The transcription factor STAT5 is constitutively active in several hematological malignancies and plays an essential role in CML pathogenesis [40]. CML cells are characterized by the BCR/ABL1 fusion gene, which generates a constitutively activated tyrosine kinase responsible for malignant transformation and STAT5 activation. STAT5 has been described as indispensable in mouse models of both lymphoid and myeloid Bcr-Abl-dependent leukemia, since STAT5 deletion suppressed initiation and maintenance of these types of cancer [41].

The absence of STAT5 modulation in K562 cells suggests cell line-specific regulatory mechanisms, potentially reflecting baseline STAT5 activity, BCR-ABL signaling, or differential engagement of downstream targets. Although STAT5 is commonly described as constitutively activated in CML, this feature is not universal, as its activation has been shown to vary across different cell lines and to remain cytokine-dependent in certain hematopoietic models [42]. This variability may contribute to the differential responsiveness observed between K562 and MOLM-6 cells. In K562 cells, STAT5 is known to be constitutively activated due to BCR-ABL signaling, which may reduce its susceptibility to further modulation. In contrast, MOLM-6 cells may retain a more dynamic regulation of STAT5 activity, making this pathway more responsive to external perturbations. Importantly, our observations reflect changes in STAT5 protein expression or stability rather than its activation status, as only total STAT5 protein levels were evaluated. Since phosphorylated STAT5 (p-STAT5) was not assessed, no conclusions can be drawn regarding STAT5 activation or downstream signaling activity.

However, given the limited characterization of MOLM-6 in the current literature, these interpretations remain speculative and warrant further investigation.

STAT5 leads to the expression of proteins involved in cell survival promotion and cell cycle progression, such as anti-apoptotic Bcl-2 family members and cyclins D [43, 44]. STAT5 expression is necessary for maintaining Bcl-2 family gene expression and mitochondrial membrane stability [45]. In previous studies, it was shown that a STAT5 consensus element is present in the Bcl-2 gene promoter and that Bcl-2 expression can be induced by STAT5 [46, 47]. Although a decrease in Bcl-2 expression has been described in STAT5-silenced Ph + ALL cells and in STAT5-deficient mice and primary mast cells [44, 45, 48], in treated MOLM-6 cells Bcl-2 expression remained unchanged despite STAT5 downregulation. This result suggests that other members of the Bcl-2 family proteins could be targets of STAT5 in this type of leukemic cells, such as the antiapoptotic proteins Bcl-X_L_ and Mcl-1 or the proapoptotic Bim. In Ph + ALL cells, STAT5 silencing, in addition to downregulating Bcl-2, also resulted in decreased antiapoptotic Mcl-1 expression and upregulation of the pro-apoptotic protein Bim [44]. Furthermore, it has been demonstrated that Bcl-X_L_ expression is dependent on STAT5 activity in Bcr-Abl-expressing cell lines and cells from CML patients [49] and Bcl-X promoters contain STAT binding sites to which STAT5 constitutively binds in Bcr-Abl transformed cells [50].

To validate the role of STAT5 downregulation in lobaric- and rhizocarpic acid-induced apoptosis in MOLM-6 cells, further investigations employing STAT5 knockdown or treatment with specific inhibitors are required. These approaches may yield comparable effects to those observed in this study and better elucidate the underlying molecular mechanism.

A limitation of the present study is the absence of full dose-response characterization and IC₅₀ determination in MOLM-6 cells. Consequently, direct quantitative comparison of drug sensitivity between K562 and MOLM-6 models cannot be conclusively established. The concentrations used should be considered exploratory and selected to induce measurable biological effects suitable for mechanistic investigation rather than representing normalized pharmacological stress across cell types. Future studies incorporating complete dose-response analyses will be necessary to define relative potency and therapeutic windows.

In conclusion, this study provides evidence that lobaric and rhizocarpic acids modulate proliferation, apoptosis, and cell cycle-related pathways in two leukemic cell models under defined experimental conditions. Differences in response kinetics likely reflect intrinsic cellular features, proliferation rates, and pathway-specific susceptibilities, including STAT5 signaling.

These findings provide new insights into biological activity of lichen-derived metabolites and support further investigation of lobaric and rhizocarpic acids as potential modulators of leukemic cell survival. Future studies, including extended dose–response analyses, evaluation of potential synergic effects, selectivity assessment in non-malignant cells, and in vivo validation will be required to fully characterize their therapeutic potential.

## Supplementary Information

Below is the link to the electronic supplementary material.

Supplementary Material 1

Supplementary Material 2

## Data Availability

Data associated with this work are available from the corresponding author upon reasonable request.
