# Supplementary material for: Pro-apoptotic and cell cycle-modulating effects of lobaric and rhizocarpic acids in human leukemic cell lines
Source: Mol Biol Rep. 2026 Jun 23;53(1):977. doi: 10.1007/s11033-026-12179-x (PMC13290786; doi:10.1007/s11033-026-12179-x)
Supplement: Supplementary file 1 — Supplementary Material 1 [file 11033_2026_12179_MOESM1_ESM.docx]

Figure S1

**Fig. 1** Investigated lichen secondary metabolites
