# Supplementary material for: Pro-apoptotic and cell cycle-modulating effects of lobaric and rhizocarpic acids in human leukemic cell lines
Source: Mol Biol Rep. 2026 Jun 23;53(1):977. doi: 10.1007/s11033-026-12179-x (PMC13290786; doi:10.1007/s11033-026-12179-x)
Supplement: Supplementary file 2 — Supplementary Material 2 [file 11033_2026_12179_MOESM2_ESM.docx]

**Figure S1.** Effect of lobaric and rhizocarpic acid treatments for 48 h on K562 cell survival. Cellular viability was determined by the MTT assay. The results represent the mean ± SD of three independent experiments.

*p < 0.05, **p < 0.01 and ***p < 0.001 compared to the untreated control.
